# Attractive and repulsive residue fragments at the interface of SARS-CoV-2 and hACE2

**DOI:** 10.1038/s41598-021-91877-x

**Published:** 2021-06-15

**Authors:** Jorge H. Rodriguez

**Affiliations:** Computational Biomolecular Physics Group, Department of Physics and Astronomy, Purdue University, West Lafayette, IN 47907-2036 USA

**Keywords:** Biophysics, Molecular biophysics, Structural biology, Molecular modelling, Virology, SARS-CoV-2

## Abstract

The initial stages of SARS-CoV-2 coronavirus attachment to human cells are mediated by non-covalent interactions of viral spike (S) protein *receptor binding domains* (S-RBD) with human ACE2 receptors (hACE2). Structural characterization techniques, such as X-ray crystallography (XRC) and cryoelectron microscopy (cryo-EM), previously identified SARS-CoV-2 spike protein conformations and their surface residues in contact with hACE2. However, recent quantum-biochemical calculations on the structurally related S-RBD of SARS-CoV-1 identified some contact-residue fragments as intrinsically attractive and others as repulsive. This indicates that not all surface residues are equally important for hACE2 attachment. Here, using similar quantum-biochemical methods, we report some four-residue fragments (i.e *quartets*) of the SARS-CoV-2 S-RBD as intrinsically attractive towards hACE2 and, therefore, directly promoting host–virus non-covalent binding. Other fragments are found to be repulsive although involved in intermolecular recognition. By evaluation of their respective intermolecular interaction energies we found two hACE2 fragments that include contact residues (ASP30, LYS31, HIS34) and (ASP38, TYR41, GLN42), respectively, behaving as important SARS-CoV-2 attractors. LYS353 also promotes viral binding *via* several mechanisms including *dispersion* van der Waals forces. Similarly, among others, three SARS-CoV-2 S-RBD fragments that include residues (GLN498, THR500, ASN501), (GLU484, PHE486, ASN487) and (LYS417), respectively, were identified as hACE2 attractors. In addition, key hACE2 *quartets* identified as weakly-repulsive towards the S-RBD of SARS-CoV-1 were found strongly attractive towards SARS-CoV-2 explaining, in part, the stronger binding affinity of hACE2 towards the latter coronavirus. These findings may guide the development of synthetic antibodies or identify potential viral epitopes.

## Introduction

The family of coronaviruses includes the genetically and structurally related, although not identical, SARS-CoV-1 and SARS-CoV-2^1–4^. The former, a potentially reemerging pathogen^5,6^, initiated an infectious outbreak in late 2002 whereas the latter is responsible for the current worldwide pandemic. It has been realized that specific structural differences between surface proteins of each coronavirus requires, also specific, investigation of the binding mechanism of each virus to host human cells^7^. As such, molecular-level understanding of the binding mechanisms of each coronavirus, such as SARS-CoV-1 or SARS-CoV-2, to human cells is important for developing effective countermeasures including antiviral drugs and vaccines. Here, we characterize the interaction-energy profile of the interface between the *receptor binding domains* of SARS-CoV-2 spike (S) proteins (S-RBD) and their interacting host cell receptors. This allows us to identify the specific, host and viral, residue fragments which are mainly responsible for host-virus attachment.

Structural characterization techniques, including X-ray crystallography (XRC) and cryogenic electron microscopy (cryo-EM), provide molecular-level insight about the structural basis of viral infectivity. The prefusion conformation (*up*-state) of SARS-CoV-2 spike proteins has been reported^8^ from cryo-EM studies. The human angiotensin-converting enzyme 2 (hACE2), a receptor at the outer surface of host cells, has been identified as a point of entry for both coronaviruses, SARS-CoV-1^9,10^ and SARS-CoV-2^11–13^. However, the attachment mechanisms of each coronavirus to hACE2 are not identical due, in part, to structural differences of their respective S-RBDs. XRC and/or cryo-EM techniques have also identified interacting (i.e. contact) residues at the interface of each coronavirus, SARS-CoV-1^1^ and SARS-CoV-2^14–17^, with the hACE2 receptor. Nevertheless, although contact residues have been identified and structurally characterized, their relative importance for host-virus attachment remains somewhat unclear. This is due to the specific, and often different, structural and biophysical properties of each contact residue which, in some cases, makes them either attractive or repulsive. Therefore, structural closeness between host and viral residues does not necessarily correlate with their effectiveness as intermolecular attractors which promote intermolecular attachment.

Computational methods can use the structural information provided by XRC or cryo-EM to fill gaps in molecular-level understanding of coronavirus binding to hACE2. Recent studies, mostly based on free-energy force-field molecular dynamics, reported properties of SARS-CoV-2 residues and their host-binding mechanisms^18–20^. Some studies, including *ab initio* fragment molecular orbital (FMO) calculations^21^, identified individual residues considered important for intermolecular recognition and binding at the hACE2...S-RBD interface. An alternative methodology, implemented in the context of fragment-based quantum-biochemical calculations^7^, provides complementary insight about host-virus interactions and specifies the neutral, attractive or repulsive nature of particular hACE2 and S-RBD *fragments*. The method combines density functional calculations with van der Waals *dispersion* contributions^22^ to establish a relationship between the molecular structure of host-virus interface *fragments* and their corresponding, attractive or repulsive, interaction energies^7^. Here we used this fragment-based approach with two recent XRC structures of the SARS-CoV-2 spike protein in complex with hACE2^14,15^. These XRC structures are structurally similar at the host-virus interface and correspond to similar, thermodynamically-favorable, conformations.

For the hACE2...SARS-CoV-1 complex, contact residue fragments, some of attractive character and some of repulsive nature, were recently identified by means of fragment-based quantum-biochemical calculations^7^. Here, we proceed in a similar fashion to identify residue fragments, at the hACE2...SARS-CoV-2 interface, and determine their attractive or repulsive nature. Such information can contribute to greater specificity in the implementation of antiviral countermeasures since it identifies certain S-RBD fragments as potential antiviral targets or antibody epitopes.

The binding strength of the SARS-CoV-2 S-RBD towards hACE2 has been characterized by various methods including flow cytometry^23^, surface plasmon resonance (SPR)^8,15,17^ and atomic force microscopy^24^. The measurements are generally consistent with the S-RBD of SARS-CoV-2 binding hACE2 more strongly than SARS-CoV-1. Flow cytometry affinity measurements, relative to cell-associated and soluble hACE2 receptors, were reported and the SARS-CoV-2 S-RBD was found to bind hACE2-expressing 293T cells with greater affinity than the SARS-CoV-1 S-RBD^23^. Additional surface plasmon resonance (SPR) measurements produced lower ($K_D$ $\approx$ 4.7 nM) and higher ($K_D$ $\approx$ 31.6 nM) binding dissociation constants for SARS-CoV-2 and SARS-CoV-1, respectively. The smaller value of $K_D$ corresponds to a greater binding affinity between a S-RBD and hACE2. Thus the SPR results also indicate a stronger binding of SARS-CoV-2 towards hACE2. Here we present results which are consistent with and, more importantly, help to explain these experimental findings. We evaluate interaction energies for individual hACE2...S-RBD *supermolecular fragments* which support a stronger binding of the S-RBD from SARS-CoV-2 to hACE2 as compared to the corresponding binding of SARS-CoV-1.

A previous study characterized the hACE2...SARS-CoV-1 interface and determined which four-residue fragments (i.e. *quartets*) are most attractive or most repulsive^7^. It was determined that two hACE2-centered *quartets* and three S-RBD-centered *quartets* are mainly responsible for the attractive interaction of the SARS-CoV-1 S-RBD with hACE2. Here, we have studied the corresponding interactions of the SARS-CoV-2 S-RBD with hACE2 and determined *quartet* fragments of dominant attractive or repulsive nature. We have also identified individual residues that, more dominantly, promote hACE2...S-RBD attachment. In addition we have evaluated *partial* interaction energies corresponding to individual SARS-CoV-2...hACE2 *supermolecular fragments* and compared them with those corresponding to the SARS-CoV-1...hACE2 complex. We have identified key differences, in binding mechanism and interaction strength, between fragments of the two coronaviruses, relative to hACE2, which explain in part their different binding affinities.

## Results

### Biomolecular fragmentation into four-residue (*quartet*) units

**Figure 1:**
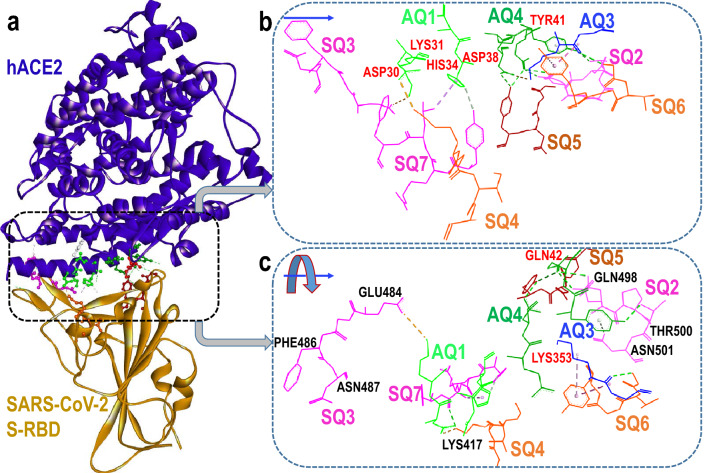
Identification of main *attractive* four-residue fragments (*quartets*) of the spike (S) protein from SARS-CoV-2 and the human (h) receptor ACE2. (**a**) Structure of the prefusion conformation of the viral spike (S) protein (shown in gold) in contact with the human angiotensin-converting enzyme 2 (hACE2) (shown in blue)^14^. The spike protein *receptor binding domain* (S-RBD) makes contact with hACE2 residues. The hACE2-S-RBD interface is enclosed in the dashed box and some four-residue (i.e *quartet*) fragments, which promote host-virus attraction, are shown. (**b**) Magnified view of three hACE2 *quartets*, **AQ1, AQ3** and **AQ4**, involved in significant attractive interactions with the S-RBD. Six S-RBD *quartets*, **SQ2, SQ3, SQ4, SQ5, SQ6** and **SQ7**, displaying attractive character towards hACE2 are also shown. A rotated view is presented in (**c**). Labels of selected *attractive* residues corresponding to hACE2 (in red) and the S-RBD (in black) are included. All constituent residues of each *quartet* are listed in Tables 1, 2.

**Figure 2:**
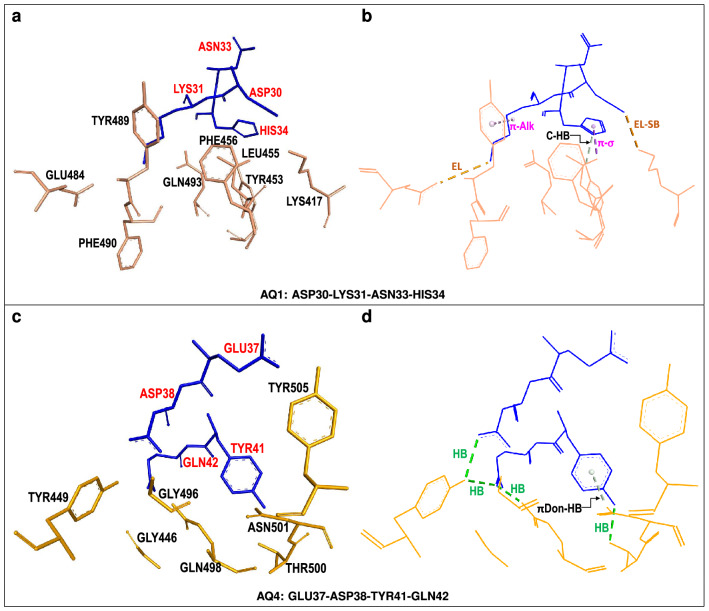
Two hACE2-centered *supermolecular fragments* giving rise to attractive hACE2...S-RDB interactions. (**a**,**c**) hACE2 *quartet* residues (shown in blue) and neighboring SARS-CoV-2 S-RBD residues (shown in gold) corresponding to the dominant *attractive* host-virus interaction energies. (**b**,**d**) Selected intermolecular interactions corresponding to (i) conventional hydrogen bonds (HB) (dotted green lines), (ii) carbon (non-classical) (C-HB) or $\pi$-donor hydrogen bonds ($\pi$Don-HB) (dotted silver lines), (iii) $\pi$-$\sigma$ or $\pi$-alkyl ($\pi$-Alk) interactions (dotted pink lines) and (iv) electrostatic interactions (EL) including a salt bridge (EL-SB) (brown dotted lines).

The hACE2...S-RBD structure considered in this work is shown in Fig. 1. To identify portions of host and viral surface proteins, most directly responsible for their attachment, we followed the fragmentation into four-residue units (i.e. *quartet*) methodology^7^. These protein sub-structures are small enough to provide a degree of residue specificity and also large enough to incorporate neighboring residue interactions which render their use, in quantum-biochemical interaction energy calculations, meaningful. Although numerically possible, fragmentation into units of less than four residues may not incorporate sufficient single-residue structural environments to properly mimic their intra- and inter-molecular interactions within a protein environment. In this work, each hACE2 or S-RBD *quartet* and their neighboring S-RBD or hACE2 residues, respectively, (within a 4.5 $\AA$ radius of any *quartet* non-hydrogen atom) is defined as a *supermolecular fragment* with two examples shown in Fig. 2.

**Table 1:** hACE2 *quartets* and their interaction energies [kcal/mol]^a^ with neighboring^b^ SARS-CoV-2 S-RBD residues.

*Quartet*	Human ACE2 receptor residues	$E_{Int}^{DFT}$	$E_{Int}^{DD}$	$E_{Int}^{Total}$
**AQ1**	ASP30-LYS31-ASN33-HIS34	− 58.31	− 26.36	− 84.67
**AQ2**	GLN24-ALA25-LYS26-THR27	+ 41.22	− 15.90	+ 25.32
**AQ3**	GLU329-ASN330-LYS353-GLY354	+ 26.79	− 21.95	+ 4.84
**AQ4**	GLU37-ASP38-TYR41-GLN42	− 45.53	− 16.58	− 62.11
**AQ5**	MET82-TYR83-GLN89-ASN90	+ 35.43	− 10.31	+ 25.12
**AQ6**	SER44-LEU45-ALA46-SER47	+ 27.63	− 1.56	+ 26.06
**AQ7**	SER77-THR78-LEU79-ALA80	+ 27.80	− 2.42	+ 25.37

^a^DFT energies computed at 6-311+G(d,p)/B3LYP level; *Dispersion* (DD) corrections evaluated with semiempirical method^22^.

^b^S-RBD residues within 4.5 $\AA$ of hACE2 *quartet* non-hydrogen atoms included.

The present quantum-biochemical approach calculates interaction energies, as defined by Eqs. (1), (2), and includes intermolecular interactions in the low temperature limit. The methodology is based on all-electron calculations and does not distinguish interactions according to conventional (e.g. force-field) classifications, with the exception of *dispersion* van der Waals corrections^7,22^. However, qualitatively, it is possible to establish correlations between the current all-electron calculations and results from other methods which identify interactions such as hydrogen bonding and other electrostatic mechanisms.

### Identification of key hACE2 *quartet* interactions with the S-RBD of SARS-CoV-2

**Figure 3:**
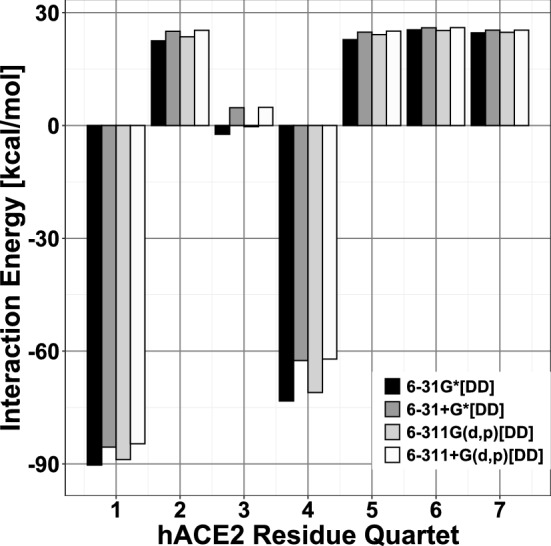
Interaction energies between hACE2 *quartets* and neighboring SARS-CoV-2 S-RBD residues. Interactions of repulsive (positive) and attractive (negative) character [kcal/mol] between hACE2 *quartets* and SARS-CoV-2 S-RBD residues within a 4.5 $\AA$ radius. For each *quartet* four adjacent vertical bars are shown corresponding to density functional calculations with 6-31G*, 6-31+G*, 6-311G(d,p) and 6-311+G(d,p) basis sets, respectively, plus additional van der Waals *dispersion* [DD]^22^ corrections. Results from different basis sets are qualitatively similar. *Quartet* and *supermolecular fragment* coordinates used in calculations taken from PDB entry 6LZG.

Table 1 and Fig. 3 display *partial* energies corresponding to hACE2 *quartets* interacting with their respective neighboring S-RBD residues. We notice that two hACE2 *quartets*, **AQ1** (ASP30-LYS31-ASN33-HIS34) and **AQ4** (GLU37-ASP38-TYR41-GLN42), are strongly attractive towards the S-RBD and display corresponding interaction energies of − 84.67 and − 62.11 kcal/mol. Figure 2 shows the respective *supermolecular fragments* along with their constituent residues.

Qualitatively, Fig. 2 illustrates selected non-covalent interactions between hACE2 and S-RBD residues. In particular, it shows the important roles of some five- or six-membered aromatic rings. The imidazole ring of hACE2[HIS34] participates in a $\pi$-sigma interaction with S-RBD[LEU455] whereas the phenol ring of S-RBD[TYR489] is involved in a $\pi$-alkyl interaction with hACE2[LYS31] (Fig. 2b). Similarly, hACE2[TYR41] participates in $\pi$-donor hydrogen bonding with S-RBD[ASN501] (Fig. 2d). More fundamentally, these aromatic-ring-related mechanisms are largely incorporated in corresponding van der Waals *dispersion* contributions to partial interaction energies which, for **AQ1** and **AQ4**, were on the order of − 26 and − 17 kcal/mol, respectively (Table 1). In addition, the presence of conventional hydrogen bonds is noticed in the **AQ4**-centered fragment whereas other electrostatic interactions play a role in the **AQ1**-centered fragment.

**Figure 4:**
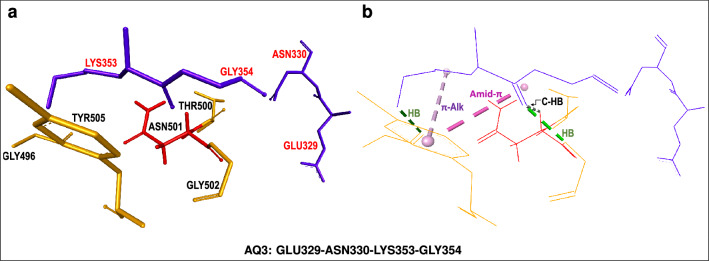
hACE2-centered *supermolecular fragment* giving rise to mixed, attractive and repulsive, hACE2...S-RDB interactions. (**a**) hACE2 *quartet* residue (shown in blue) and neighboring SARS-CoV-2 S-RBD residues (shown in gold). S-RBD[ASN501] is shown in red. (**b**) Selected intermolecular interactions corresponding to (i) conventional hydrogen bonds (HB) (dotted green lines), (ii) carbon (non-classical) hydrogen bond (C-HB) between LYS353 and ASN501 (dotted silver lines) and (iii) $\pi$-alkyl ($\pi$-Alk) or amide-$\pi$ interactions (dotted pink lines).

The **AQ3**-centered fragment (Fig. 4) displays a mixed set of interactions and, accordingly, its interaction energy includes repulsive (+ 26.79 kcal/mol) and attractive (− 21.95 kcal/mol) contributions. This fragment includes hACE2[LYS353] which interacts in multiple ways, promoting attraction, with its neighboring S-RBD residues. This residue undergoes $\pi$-sigma and $\pi$-alkyl interactions with the aromatic ring of S-RBD[TYR505] in addition to conventional hydrogen bonding with S-RBD residues GLY496 and GLY502. Finally, hACE2[LYS353] also interacts, *via* non-classical (carbon) hydrogen bonding with S-RBD[ASN501]. Thus hACE2[LYS353] is an important contact residue which promotes non-covalent binding to the viral spike protein through several and simultaneous mechanisms.

**Table 2:** SARS-CoV-2 S-RBD *quartets* and their interaction energies [kcal/mol]^a^ with neighboring^b^ hACE2 residues.

*Quartet*	SARS-CoV-2 S-RBD residues	$E_{Int}^{DFT}$	$E_{Int}^{DD}$	$E_{Int}^{Total}$
**SQ1**	GLN493-SER494-TYR495-GLY496	+ 59.56	− 7.74	+ 51.82
**SQ2**	GLN498-PRO499-THR500-ASN501	+ 12.67	− 19.96	− 7.29
**SQ3**	GLU484-GLY485-PHE486-ASN487	− 17.84	− 17.37	− 35.21
**SQ4**	GLY416-LYS417-ILE418-ALA419	− 57.67	− 1.50	− 59.18
**SQ5**	GLY446-GLY447-ASN448-TYR449	− 7.61	− 3.60	− 11.21
**SQ6**	GLY502-VAL503-GLY504-TYR505	+ 5.71	− 15.00	− 9.28
**SQ7**	TYR453-ARG454-LEU455-PHE456	+ 49.40	− 17.51	+ 31.89
**SQ8**	TYR473-GLN474-ALA475-GLY476	+ 24.69	− 5.32	+ 19.37
**SQ9**	TYR489-PHE490-PRO491-LEU492	+ 67.24	− 8.25	+ 58.99

^a^DFT energies computed at 6-311+G(d,p)/B3LYP level; *Dispersion* (DD) corrections evaluated with semiempirical method^22^.

^b^hACE2 residues within 4.5 $\AA$ of S-RBD *quartet* non-hydrogen atoms included.

### Identification of key SARS-CoV-2 S-RBD *quartet* interactions with hACE2

**Figure 5:**
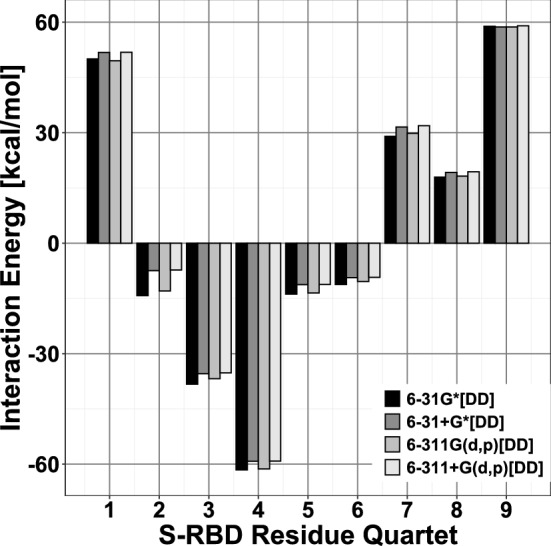
Interaction energies between SARS-CoV-2 S-RBD *quartets* and neighboring hACE2 residues. Interactions of repulsive (positive) and attractive (negative) character [kcal/mol] between SARS-CoV-2 *quartets* and hACE2 residues within a 4.5 $\AA$ radius. For each *quartet* four adjacent vertical bars are shown corresponding to density functional calculations with 6-31G*, 6-31+G*, 6-311G(d,p) and 6-311+G(d,p) basis sets, respectively, plus additional van der Waals *dispersion* [DD]^22^ corrections. Results from different basis sets are qualitatively similar. *Quartet* and *supermolecular fragment* coordinates used in calculations taken from PDB entry 6LZG.

**Figure 6:**
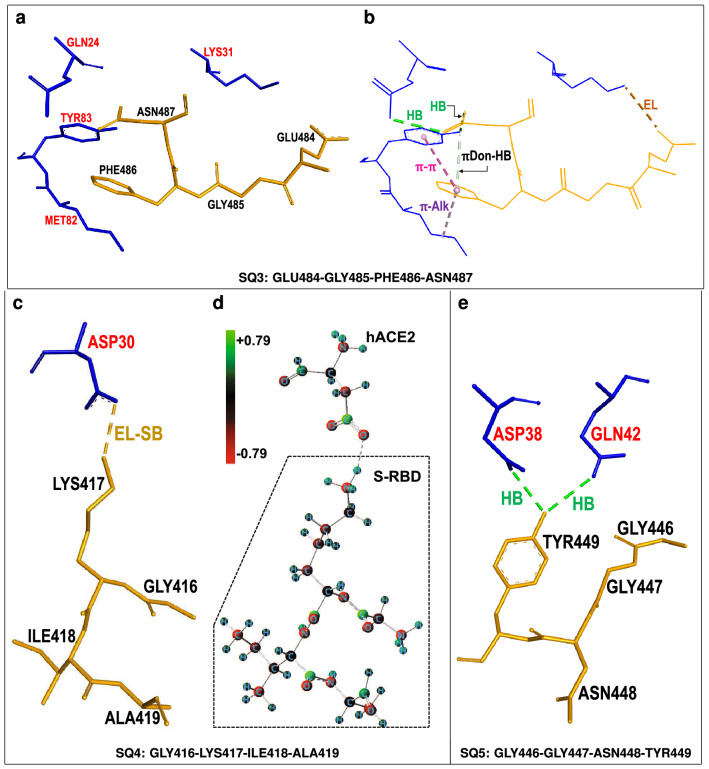
Three S-RBD-centered *supermolecular fragments* giving rise to attractive hACE2...S-RDB interactions. (**a**,**c**,**e**) SARS-CoV-2 S-RBD *quartet* residues (shown in gold) and neighboring hACE2 residues (shown in blue) corresponding to dominant *attractive* host-virus interactions. (**b**,**c**,**e**) Selected intermolecular interactions corresponding to (i) conventional hydrogen bonds (HB) (dotted green lines), (ii) $\pi$-donor (non-classical) hydrogen bonds ($\pi$Don-HB) (dotted silver lines), (iii) $\pi$-$\pi$ or $\pi$-alkyl ($\pi$-Alk) interactions (dotted pink lines) and (iv) electrostatic (EL) interactions including a salt bridge (EL-SB) (brown dotted lines). (**d**) Distribution of partial charges, calculated with NBO method^25^, corresponding to positively charged (green spheres) and negatively charged (red spheres) atoms of **SQ4**-centered fragment.

Table 2 and Fig. 5 display *partial* interaction energies corresponding to S-RBD *quartets* interacting with their respective neighboring hACE2 residues. There are several S-RBD *quartets* of attractive nature towards hACE2 with two, **SQ3** (GLU484-GLY485-PHE486-ASN487) and **SQ4** (GLY416-LYS417-ILE418-ALA419), being dominant. The attractive interaction energies of these two *quartets*, respectively on the order of − 35 and − 59 kcal/mol, have fairly different compositions with **SQ3** incorporating a substantial contribution from *dispersion* (about − 17 kcal/mol) and **SQ4** mostly due to conventional electrostatic mechanisms including hydrogen bonding. Figure 6a,c show the molecular structures of the corresponding *supermolecular fragments* whereas selected qualitative interactions are displayed in Fig. 6b,d. The latter illustrate the different physico-chemical origins of each *quartet*’s attractive nature. The **SQ3**-centered fragment displays a complex combination of intermolecular mechanisms including the aromatic ring of S-RBD[PHE486] undergoing simultaneous $\pi$-alkyl and $\pi$-$\pi$ stacked interactions with hACE2[MET82] and hACE2[TYR83], respectively. These interactions, in turn, are reflected in the sizable *dispersion* contribution (about − 17 kcal/mol) of the fragment interaction energy (Table 2). In addition, **SQ3** forms conventional (dotted green lines) and non-conventional (dotted silver lines) hydrogen bonds with hACE2 residues and undergoes other electrostatic interactions (dotted yellow lines). Figure 6d displays partial charges for the **SQ4**-centered fragment with atoms colored according to the magnitude and sign of their respective positive (green) or negative (red) charges. The atomic charges illustrate the mostly electrostatic origin of this fragment’s attractive interaction energy. In particular, the positively charged atoms of S-RBD[LYS417] exert attraction on the strongly negatively charged atoms of hACE2[ASP30] *via* both, conventional and hydrogen bonding, electrostatic interactions. Figure 6e shows the weakly attractive **SQ5**-centered fragment, with interaction energy of about − 11 kcal/mol, whereby S-RBD[TYR449] forms hydrogen bonds with hACE2 residues ASP38 and GLN42.

**Figure 7:**
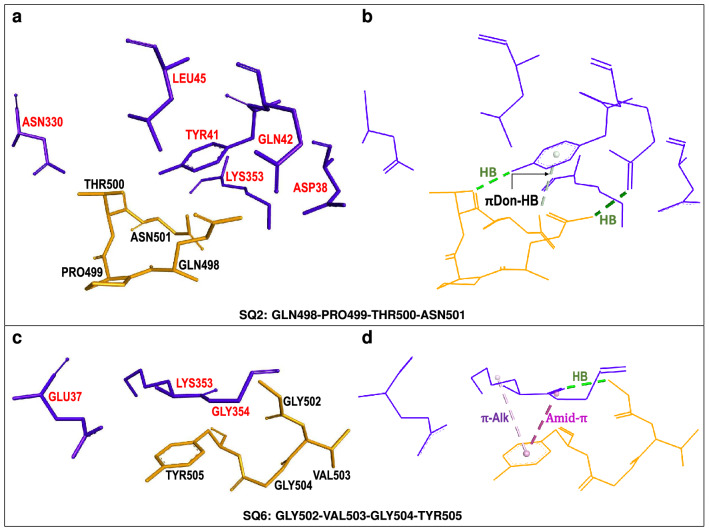
Two S-RBD-centered *supermolecular fragments* giving rise to attractive hACE2...S-RDB interactions. (**a**,**c**) SARS-CoV-2 S-RBD *quartet* residues (shown in brown) and neighboring hACE2 residues (shown in blue) corresponding to weakly *attractive* host–virus interactions. (**b**,**d**) Selected intermolecular interactions corresponding to (i) conventional hydrogen bonds (HB) (dotted green lines), (ii) $\pi$-donor (non-classical) ($\pi$Don-HB) hydrogen bonds (dotted silver lines) and (iii) $\pi$-alkyl ($\pi$-Alk) or amide-$\pi$ interactions (dotted pink lines).

Table 2 and Fig. 7a,c show two other S-RBD *quartets*, **SQ2** and **SQ6**, which despite being only weakly attractive, incorporate substantial attractive *dispersion* contributions. The latter, as illustrated by Fig. 7b,d, are associated with aromatic groups of contact residues interacting in various ways. S-RBD[ASN501] undergoes $\pi$-donor hydrogen bonding with the phenol ring of hACE2[TYR41] whereas the phenol ring of S-RBD[TYR505] simultaneously interacts, *via* $\pi$-alkyl and amide-$\pi$ stacked mechanisms, with hACE2 residues LYS353 and GLY354, respectively. For both *quartets* these attractive interactions are partially counteracted by repulsive terms leading to their attractive, but relatively weak, interaction energies.

We carried out calculations with two different X-ray crystallographic structures corresponding to hACE2 in complex with the prefusion conformation of SARS-CoV-2 (PDB entries 6LZG and 6M0J)^14,15^. Interaction energies obtained with both PDB entries were remarkably similar save minor exceptions related to the viral environment of *quartet* **AQ6** as explained in the Methods section. This indicates that both X-ray structures correspond to very similar thermodynamically-favorable hACE2...S-RBD conformations. To ensure quantitative and qualitative consistence, all interaction energies were computed, independently, with four different basis sets of increasing size [6-31G*, 6-31+G*, 6-311G(d,p) and 6-311+G(d,p)]. Figures 3 and 5 (based on PDB entry 6LZG) show qualitatively consistent results for all basis sets. Similar results (based on PDB entry 6M0J) are given in Supplementary Figs. S1, S2. Tables 1, 2 show results obtained with the 6-311+G(d,p) basis and Supplementary Tables S1–S3, S5–S7 display qualitatively consistent results for the other basis sets.

## Discussion

### *Attractive* residues at the interface of hACE2 with the S-RBD of SARS-CoV-2

Following the procedure outlined by Rodriguez and Gupta^7^ we identified individual residues involved in intermolecular host-virus attraction. Although the main quantitative output of the present calculations allows the identification of *quartet* fragments, of either attractive or repulsive nature, it is also possible to identify key residues based on qualitative analysis of their parent fragments. Establishing correlations between the two sets of calculations reported in this work, whereby (i) hACE2 *quartets* interact with S-RBD residues and (ii) S-RBD *quartets* interact with hACE2 residues, permits the identification of individual residues involved in attractive interactions which promote host-virus attachment.

More specifically, **AQ1** residues ASP30, LYS31 and HIS34 as well as **AQ4** residues ASP38, TYR41 and GLN42 were identified as constituent members of attractive *quartets* in both sets of calculations. These hACE2 residues are likely important attractors of their respective S-RBD contacts. In addition, residue LYS353 is involved in attractive interactions with the S-RBD even though its parent *quartet* (**AQ3**) displays a weakly-repulsive character according to its interaction energy (+ 4.84 kcal/mol). Qualitative analysis indicates that, consistent with the attractive *dispersion* energy component of its parent fragment (− 21.95 kcal/mol), hACE2[LYS353] undergoes several favorable interactions with the SARS-CoV-2 S-RBD which promote host-virus attachment. Namely, LYS353 interacts *via* conventional hydrogen bonding with S-RBD[GLY496,GLY502] and *via* non-classical (i.e. carbon) hydrogen bonding with S-RBD[ASN501]. In addition, LYS353 undergoes $\pi$-alkyl and amid-$\pi$-stacked interactions with the aromatic ring of S-RBD[TYR505]. Thus, although within the present framework it belongs to a weakly-repulsive *quartet*, by itself LYS353 is an important, perhaps crucially important, attractor of the SARS-CoV-2 S-RBD. We notice that hACE2[LYS353] was also identified as an important attractor towards the S-RBD of SARS-CoV-1^7^.

A similar analysis provides insight about individual S-RBD residues involved in attractive interactions. **SQ2** residues GLN498, THR500 and ASN501; **SQ3** residues GLU484, PHE486 and ASN487; **SQ4** residue LYS417, **SQ5** residue TYR449 as well as **SQ6** residue TYR505 all participate in attractive interactions, in both sets of calculations, highlighting their roles in promoting binding to hACE2. Residues of three additional, nominally repulsive, S-RBD *quartets* should also be mentioned. First, **SQ1**(GLY496) attracts hACE2[LYS353] *via* hydrogen bonding. Second, although **SQ7** produces a net repulsive interaction (+ 31.89 kcal/mol) towards hACE2, it incorporates a minority, but sizable, attractive *dispersion* contribution (− 17.5 kcal/mol). The latter arises from attractive interactions of S-RBD residues LEU455 and TYR453 with the aromatic ring of hACE2[HIS34], consistent with the calculated *dispersion* component. More specifically, these interactions correspond to $\pi$-sigma and non-classical (i.e. carbon) hydrogen bonding mechanisms, respectively. Third, a similar situation occurs with S-RBD *quartet* **SQ9** which, being overall of repulsive character, includes the attractive residue TYR489 undergoing $\pi$-alkyl interactions with hACE2[LYS31]. This is consistent with the minority (− 8.25 kcal/mol) *dispersion* interaction of the *quartet*.

The identification of some attractive hACE2 residues, based on analysis of *quartet* interaction energies, is consistent with prior structural studies. In particular hACE2 sites 31 and 353, corresponding to LYS31 and LYS354, were also considered important binding hotspots^26^ with favorable viral interactions that aid in the process of intermolecular recognition. Nevertheless, structural analysis alone is not able to capture the combined action of multiple fragment-to-fragment or residue-to-residue interactions which are, by contrast, carefully quantified in the present quantum-biochemical calculations. The latter, being all-electron in nature, take into account even subtle variations in fragment structure, atomic number of constituent atoms and interatomic distances. For example, previous structural considerations suggest that S-RBD residue GLN493 is critical and favors hACE2 attraction^26^. However the present results indicate that GLN493, consistent with its longer distance to hACE2 residue LYS31, is not as important as S-RBD GLY496 which, consistent with its shorter distance to hACE2 residue LYS353, is herein identified as a more important attractor.

### Comparison of SARS-CoV-1 and SARS-CoV-2 interactions with hACE2

The interplay of hACE2 residues with SARS-CoV-1, also based on a *quartet* fragment methodology, was recently reported^7^. This allows for a direct comparison of the dominant interactions of hACE2 with the S-RBDs of SARS-CoV-1 and SARS-CoV-2. Such a comparison reveals similarities but also important differences which help to explain hACE2...S-RBD binding affinities measured by several groups^8,15,17,23,24^, for the two viruses. We found that hACE2 *quartet* **AQ4** (GLU37-ASP38-TYR41-GLN42) is strongly attractive towards both viral S-RBDs with interaction energies of approximately − 55 and − 62 kcal/mol for SARS-CoV-1^7^ and SARS-CoV-2 (Table 1), respectively. For this *quartet* the attractive interaction energy is about 11% stronger in the latter case indicating that it binds more strongly to the S-RBD of SARS-CoV-2. This contributes, in part, to the reported stronger hACE2 binding to SARS-CoV-2^8,15,17,23,24^. A comparison of the makeup of the interaction energies of **AQ4** with the S-RBDs of the two coronaviruses indicates some similarities. Namely, both incorporate roughly the same contributions from van der Waals *dispersion* (about − 16 kcal/mol) which are related to intermolecular interactions of the TYR41 aromatic ring. We stress, however, that some of the neighboring S-RBD residues that interact, within the same radius, with **AQ4** are different in type and number for the two coronaviruses.

Whereas hACE2 *quartet* **AQ3** (GLU329-ASN330-LYS353-GLY354) was found to be strongly attractive towards SARS-CoV-1^7^, the same is not true with respect to SARS-CoV-2 despite the fact that in both cases there are significant attractive contributions from *dispersion*. This is because in the interaction with SARS-CoV-1 there are additional attractive contributions that enhance the binding character of **AQ3**. By contrast, the interaction with SARS-CoV-2 includes significant repulsive contributions that yield **AQ3** as slightly repulsive.

**Figure 8:**
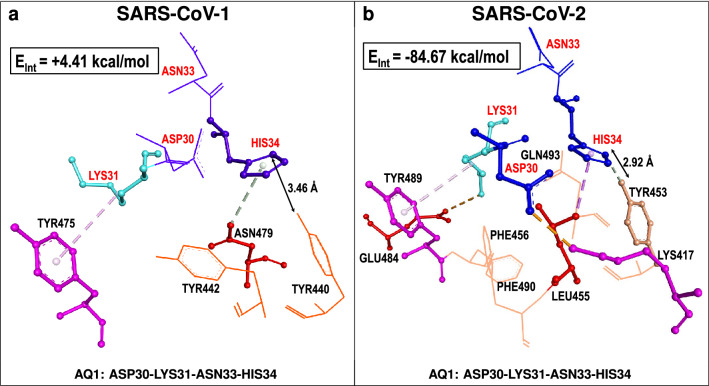
Comparison of hACE2-centered *supermolecular fragment* corresponding to hACE2...S-RDB interfaces of SARS-CoV-1 and SARS-CoV-2. Residues of hACE2 *quartet* **AQ1** (red labels) and neighboring S-RBD residues (black labels) corresponding to (**a**) SARS-CoV-1 and (**b**) SARS-CoV-2 are shown. Selected intermolecular interactions corresponding to (i) carbon (non-classical) hydrogen bonds (dotted silver lines), (ii) $\pi$-sigma or $\pi$-alkyl interactions (dotted pink lines) and (iii) electrostatic interactions (dotted brown lines). Within the same 4.5 $\AA$ radius **AQ1** interacts, *via* several attractive mechanisms, with more contact residues of SARS-CoV-2 than residues of SARS-CoV-1. Total interaction energies for each *supermolecular fragment* ($E_{Int}^{Total}$) are shown^7^ (Table 1).

The opposite behavior was observed for hACE2 *q*uartet **AQ1** (ASP30-LYS31-ASN33-HIS34) which was weakly repulsive towards SARS-CoV-1 (+ 4.41 kcal/mol)^7^ but strongly attractive towards SARS-CoV-2 (− 84.67 kcal/mol). In the latter case, as shown in Table 1, attractive *dispersion* contributions (− 26.34 kcal/mol) are significant and additional electrostatic attractions are even stronger (− 58.31 kcal/mol). Thus, the relative importance of hACE2 *quartet* **AQ1**, as a potential binder to either of the two viral S-RBDs, is very different. This seems to make a most striking difference in the attachment capability of the hACE2 receptor to the spike proteins of the two coronaviruses. Namely, **AQ1** is weakly repulsive towards the S-RBD of SARS-CoV-1 but strongly attractive towards the S-RBD of SARS-CoV-2. In fact the total interaction energy of **AQ1** with SARS-CoV-2 (− 84.67 kcal/mol) is largest in magnitude when compared to corresponding energies of all studied *supermolecular fragments* for either of the two host-virus non-covalent complexes, namely hACE2...SARS-CoV-1^7^ and hACE2...SARS-CoV-2 (Tables 1, 2). The molecular structure and qualitative interactions of **AQ1** with the S-RBD from SARS-CoV-2 are shown in Fig. 2**a,b**. An additional view and comparison of **AQ1** interactions with SARS-CoV-1 and SARS-CoV-2 are shown in Fig. 8. This Figure illustrates the more complex interaction of hACE2 *quartet* **AQ1** with the S-RBD of SARS-CoV-2 including a greater number and more diverse nature of attractive interactions. Structurally, within the same 4.5 $\AA$ radius, **AQ1** interacts with more residues from SARS-CoV-2 than SARS-CoV-1. These include TYR453 at a distance of 2.92 $\AA$ by comparison to SARS-CoV-1[TYR440] at the longer, and weaker interacting, distance of 3.46 $\AA$.

## Conclusion

The binding *domains* (S-RBDs) of SARS-CoV-1 and SARS-CoV-2 are structurally related but not identical with a sequence identity of about 72–73%. Furthermore the binding *motifs* (S-RBMs) of their respective spike proteins, which are integral and functional parts of their respective S-RBDs, have an identity of only 47–48%^27,28^. Therefore, one would expect similarities as well as differences in the binding mechanisms of hACE2 with the S-RBDs of the two viruses^7^, consistent with their structural and sequence variations as well as reported differences in their binding affinities^8,15,17,23,24^.

In this work we have identified which hACE2 and SARS-CoV-2 surface protein fragments give rise to dominant intermolecular attractions as well as those involved in intermolecular repulsion. Although the attractive fragments play a key role in promoting host-virus attachment, their repulsive counterparts also play an important role in the process of intermolecular recognition^7^. The present results help to explain experimentally observed host-virus affinity differences by explicitly identifying which surface fragments are primarily responsible for the binding of prefusion (*up*-state) conformations of SARS-CoV-2 spike proteins to hACE2 receptors. The degree of importance of some contact-residue fragments, in regards to their roles in promoting host-virus attachment, has been determined and quantified *via* their respective intermolecular interaction energies. Two hACE2 fragments (**AQ1** and **AQ4**) and three SARS-CoV-2 fragments (**SQ3**, **SQ4** and **SQ5**) have been identified as principal attractors together with other lesser promoters of host-virus attachment. In addition, key individual host and viral residues have been identified together with their qualitative interaction mechanisms. For example hACE2[LYS353] was found at the center of several mechanisms which collectively attract several viral residues (Figs. 4, 7).

By virtue of using the same fragment-based quantum-biochemical method, we have established crucial similarities and differences between the binding mechanisms of the hACE2... SARS-CoV-2 complex with those previously reported for hACE2...SARS-CoV-1^7^. We found that hACE2 **AQ4** (GLU37-ASP38-TYR41-GLN42) is an important structural unit for binding to both viral spike proteins since it promotes intermolecular attraction in either case. By contrast, hACE2 **AQ3** (GLU329-ASN330-LYS353-GLY354) plays dominant attractive and binding roles towards SARS-CoV-1^7^ but does not play the same roles towards SARS-CoV-2 with the notable exception of one of its residues, namely LYS353. As a major difference in binding mechanisms we found that, for SARS-CoV-2, hACE2 **AQ1** (ASP30-LYS31-ASN33-HIS34) plays a strong and dominant attractive role in sharp contrast to its weakly-repulsive behavior towards the S-RBD of SARS-CoV-1^7^. Differences in interaction energies and mechanisms of fragment **AQ1**, relative to both coronaviruses, have been illustrated in Fig. 8.

## Methods

The fragment-based methodology used in this work follows the procedure described by Rodriguez and Gupta^7^. The method relies on fragmentation of the interacting hACE2 and S-RBD surfaces into hACE2 or S-RBD four-residue units called *quartets*. More specifically, the minimum size of a hACE2 or S-RBD fragment unit included four residues in order to capture a sufficient extent of inter-residue interactions. This unit size was assessed as sufficient to mimic the immediate protein environment and to provide qualitatively meaningful intermolecular interaction energies^7^.

For hACE2, the selection of contact residues primarily followed the list given by the SARS-CoV-1 crystallographic reference (Supplementary Table S8)^1^. This allowed us to make direct comparisons between previous findings for SARS-CoV-1^7^ and those obtained in the present work for SARS-CoV-2. Some 18 hACE2 residues were considered making contact with the RBM of the SARS-CoV-1 spike protein^1^. A corresponding contact list for SARS-CoV-2 is fairly similar with most, but not all, hACE2 residues from the previous reference included. In particular, in the hACE2...SARS-CoV-1 complex, hACE2 residue 355 is considered a contact but, following similar criteria, it is not a contact relative to SARS-CoV-2^14^. The list of contact residues corresponding to the S-RBD of SARS-CoV-2 followed Wang et al.^14^ and included some 20 residues as shown in Supplementary Table S9.

In the present work the constituent residues of each *quartet* followed a criteria that a minimum of two consecutive residues be present. For example, in *quartet* **AQ1** (ASP30-LYS31-ASN33-HIS34), contact residue 31 was paired with residue 30 and, similarly, contact residue 34 was paired with residue 33. Here, residues 30 and 33 are directly (chemically) bound to 31 and 34, respectively, so no *contact* residue remained structurally isolated. One purpose of this work was to directly compare interactions of hACE2 with the S-RBDs of SARS-CoV-1 and SARS-CoV-2. Accordingly, hACE2-centered *quartets* in this work were constructed in a manner similar to the SARS-CoV-1 reference^7^. For S-RBD-centered *quartets* we used a similar but broader criteria. Here we included four consecutive residues in all *quartets* including those considered making contact with hACE2 (Supplementary Table S9). As in the previous case, one requirement was that no contact residue be structurally isolated and, therefore, we added additional (consecutive) residues when necessary.

Once such hACE2 or S-RBD *quartets* were created their neighboring S-RBD or hACE2 residues, respectively, were added to form three-dimensional structural constructs called *supermolecular fragments*. In this work a hACE2-centered or S-RBD-centered *supermolecular fragment* is defined, respectively, as (i) a hACE2 *quartet* and all neighboring S-RBD residues within a 4.5 $\AA$ radius of any *quartet* non-hydrogen atom or (ii) a S-RBD *quartet* and all neighboring hACE2 residues within a 4.5 $\AA$ radius of any *quartet* non-hydrogen atom.

After each *supermolecular fragment* was constructed hydrogen atoms were added according to criteria implemented in Discovery Studio Visualizer^29^. In addition, to assess possible differences due to hydrogen atom positioning based on a different scheme, we did hydrogen atom optimizations on hACE2 *supermolecular fragments*. Here all heavier atoms were kept frozen and only hydrogen atoms were optimized with the PM6 semiempirical method^30^ as implemented in the Gaussian package^31^. Results obtained with both hydrogen atom positioning methods are illustrated by Supplementary Tables S1 and S4. Both Tables were obtained under the same numerical conditions with the exception that the former placed hydrogen atoms according to the method implemented in Discovery Studio Visualizer^29^ and the latter used PM6^30^ optimizations. Whereas for these two methods there were relatively minor differences in absolute values of calculated energies, their overall trends were similar (Supplementary Tables S1 and S4). With the exception of Supplementary Table S4, all other Tables presented here are based on the first hydrogen addition method.

Two recently published X-ray crystallographic structures, corresponding to hACE2 in complex with the prefusion conformation of the SARS-CoV-2 spike protein (PDB entries 6LZG and 6M0J)^14,15^, were used to create *quartet* units and their corresponding *supermolecular fragments*. With few exceptions, due to their structural similarities, the qualitative results described in this work were similar for both crystallographic structures. One exception was the S-RBD environment of hACE2 *quartet* **AQ6** (SER44-LEU45-ALA46-SER47) which, within the prescribed 4.5 $\AA$ radius, included neighboring residues in PDB entry 6LZF but not in PDB entry 6M0J. In the latter case, S-RBD residues GLN498 and THR500 were slightly beyond the prescribed range and, for methodological consistency, were not included in the calculations (Supplementary Fig. S1). All numerical results presented in this work, with the exception of Supplementary Figs. S1, S2, are based on PDB entry 6LZG.

Evaluation of interaction energies, between hACE2 or S-RBD *quartets* with their respective neighboring residues, was based on all-electron Kohn-Sham density functional theory (DFT) and the B3LYP functional^32,33^ as implemented in Gaussian 16^31^. Following reference^7^ DFT calculations were supplemented with empirical *dispersion* corrections, as prescribed by the B3LYP-DD methodology^22^, with a locally implemented computer program. The latter method incorporates contributions from attractive components of van der Waals potentials which, otherwise, are missing from raw density functional calculations. The B3LYP-DD methodology allows for fairly accurate evaluation of intermolecular interaction energies, according to Eqs. (1), (2), over a range of intermolecular distances.

1 $$\begin{aligned} E_{Int}^{DFT}=& {} E_{hACE2...S-RBD}^{DFT} - E_{hACE2}^{DFT} - E_{S-RBD}^{DFT} \end{aligned}$$

2 $$\begin{aligned} E_{Int}^{DFT-DD}=& {} E_{Int}^{DFT} + E_{Int}^{DD} \end{aligned}$$

## Supplementary Information

Supplementary Information.
