# Supplementary material for: Attractive and repulsive residue fragments at the interface of SARS-CoV-2 and hACE2
Source: Sci Rep. 2021 Jun 15;11:12567. doi: 10.1038/s41598-021-91877-x (PMC8206228; doi:10.1038/s41598-021-91877-x)
Supplement: Supplementary file 1 — Supplementary Information. [file 41598_2021_91877_MOESM1_ESM.pdf]

Supplementary Material

Attractive and repulsive residue fragments at the interface of SARS-CoV-2 and hACE2

Jorge H. Rodriguez

Computational Biomolecular Physics Group, Department of Physics and Astronomy

Purdue University, West Lafayette, Indiana 47907-2036, USA

E-mail: jhrodrig@purdue.edu

TABLE S1. **hACE2 *quartets* and their interaction energies [kcal/mol][a] with neighboring[b] SARS-CoV-2 S-RBD residues.**

Quartet	**Human ACE2 Receptor Residues**	E_{Int}^{DFT}	E_{Int}^{DD}	E_{Int}^{Total}
**AQ1**	ASP30-LYS31-ASN33-HIS34	−65.39	−24.94	−90.33
**AQ2**	GLN24-ALA25-LYS26-THR27	+37.24	−14.69	+22.55
**AQ3**	GLU329-ASN330-LYS353-GLY354	+17.98	−20.34	−2.36
**AQ4**	GLU37-ASP38-TYR41-GLN42	−57.83	−15.45	−73.29
**AQ5**	MET82-TYR83-GLN89-ASN90	+32.20	−9.38	+22.82
**AQ6**	SER44-LEU45-ALA46-SER47	+27.05	−1.56	+25.48
**AQ7**	SER77-THR78-LEU79-ALA80	+27.04	−2.36	+24.68

[a] DFT energies computed at 6-31G*/B3LYP level; *Dispersion* (DD) corrections evaluated with B3LYP-DD semiempirical method. [1]

[b] S-RBD residues within 4.5 Å of hACE2 *quartet* non-hydrogen atoms included.

TABLE S2. **hACE2 *quartets* and their interaction energies [kcal/mol][a] with neighboring[b] SARS-CoV-2 S-RBD residues.**

Quartet	**Human ACE2 Receptor Residues**	E_{Int}^{DFT}	E_{Int}^{DD}	E_{Int}^{Total}
**AQ1**	ASP30-LYS31-ASN33-HIS34	−59.16	−26.36	−85.52
**AQ2**	GLN24-ALA25-LYS26-THR27	+40.96	−15.90	+25.06
**AQ3**	GLU329-ASN330-LYS353-GLY354	+26.70	−21.95	+4.75
**AQ4**	GLU37-ASP38-TYR41-GLN42	−45.92	−16.58	−62.49
**AQ5**	MET82-TYR83-GLN89-ASN90	+35.15	−10.31	+24.84
**AQ6**	SER44-LEU45-ALA46-SER47	+27.55	−1.56	+25.99
**AQ7**	SER77-THR78-LEU79-ALA80	+27.77	−2.42	+25.35

[a] DFT energies computed at 6-31+G*/B3LYP level; *Dispersion* (DD) corrections evaluated with B3LYP-DD semiempirical method. [1]

[b] S-RBD residues within 4.5 Å of hACE2 *quartet* non-hydrogen atoms included.

TABLE S3. **hACE2 *quartets* and their interaction energies [kcal/mol][a] with neighboring[b] SARS-CoV-2 S-RBD residues.**

Quartet	**Human ACE2 Receptor Residues**	E_{Int}^{DFT}	E_{Int}^{DD}	E_{Int}^{Total}
**AQ1**	ASP30-LYS31-ASN33-HIS34	−63.90	−24.94	−88.84
**AQ2**	GLN24-ALA25-LYS26-THR27	+38.31	−14.69	+23.62
**AQ3**	GLU329-ASN330-LYS353-GLY354	+20.02	−20.34	−0.32
**AQ4**	GLU37-ASP38-TYR41-GLN42	−55.55	−15.45	−71.01
**AQ5**	MET82-TYR83-GLN89-ASN90	+33.56	−9.38	+24.18
**AQ6**	SER44-LEU45-ALA46-SER47	+26.87	−1.56	+25.31
**AQ7**	SER77-THR78-LEU79-ALA80	+27.16	−2.36	+24.80

[a] DFT energies computed at 6-311G(d,p)/B3LYP level; *Dispersion* (DD) corrections evaluated with B3LYP-DD semiempirical method. [1]

[b] S-RBD residues within 4.5 Å of hACE2 *quartet* non-hydrogen atoms included.

TABLE S4. **hACE2 *quartets* and their interaction energies [kcal/mol][a] with neighboring[b] SARS-CoV-2 S-RBD residues.**

Quartet	**Human ACE2 Receptor Residues**	E_{Int}^{DFT}	E_{Int}^{DD}	E_{Int}^{Total}
**AQ1**	ASP30-LYS31-ASN33-HIS34	−73.17	−26.55	−99.72
**AQ2**	GLN24-ALA25-LYS26-THR27	+29.65	−16.20	+13.45
**AQ3**	GLU329-ASN330-LYS353-GLY354	+19.17	−21.91	−2.75
**AQ4**	GLU37-ASP38-TYR41-GLN42	−66.01	−16.72	−82.74
**AQ5**	MET82-TYR83-GLN89-ASN90	+32.76	−9.51	+23.25
**AQ6**	SER44-LEU45-ALA46-SER47	+27.73	−1.67	+26.06
**AQ7**	SER77-THR78-LEU79-ALA80	+26.32	−2.47	+23.85

[a] Hydrogen atoms were optimized with PM6 [2] semiempirical method.

[a] DFT energies computed at 6-31G*/B3LYP level; *Dispersion* (DD) corrections evaluated with B3LYP-DD semiempirical method. [1]

[b] S-RBD residues within 4.5 Å of hACE2 *quartet* non-hydrogen atoms included.

TABLE S5. **SARS-CoV-2 S-RBD *quartets* and their interaction energies [kcal/mol][a] with neighboring[b] hACE2 residues.**

Quartet	**SARS-CoV-2 S-RBD Residues**	E_{Int}^{DFT}	E_{Int}^{DD}	E_{Int}^{Total}
**SQ1**	GLN493-SER494-TYR495-GLY496	$+57.36$	-7.33	$+50.03$
**SQ2**	GLN498-PRO499-THR500-ASN501	$+4.66$	-18.93	-14.27
**SQ3**	GLU484-GLY485-PHE486-ASN487	-22.58	-15.72	-38.29
**SQ4**	GLY416-LYS417-ILE418-ALA419	-60.26	-1.31	-61.57
**SQ5**	GLY446-GLY447-ASN448-TYR449	-10.54	-3.31	-13.85
**SQ6**	GLY502-VAL503-GLY504-TYR505	$+2.49$	-13.72	-11.24
**SQ7**	TYR453-ARG454-LEU455-PHE456	$+45.33$	-16.32	$+29.01$
**SQ8**	TYR473-GLN474-ALA475-GLY476	$+23.14$	-5.24	$+17.90$
**SQ9**	TYR489-PHE490-PRO491-LEU492	$+66.83$	-7.98	$+58.85$

[a] DFT energies computed at 6-31G*/B3LYP level; *Dispersion* (DD) corrections evaluated with B3LYP-DD semiempirical method. [1]

[b] hACE2 residues within 4.5 Å of S-RBD *quartet* non-hydrogen atoms included.

TABLE S6. **SARS-CoV-2 S-RBD *quartets* and their interaction energies [kcal/mol][a] with neighboring[b] hACE2 residues.**

Quartet	**SARS-CoV-2 S-RBD Residues**	E_{Int}^{DFT}	E_{Int}^{DD}	E_{Int}^{Total}
**SQ1**	GLN493-SER494-TYR495-GLY496	$+59.53$	-7.74	$+51.79$
**SQ2**	GLN498-PRO499-THR500-ASN501	$+12.48$	-19.96	-7.49
**SQ3**	GLU484-GLY485-PHE486-ASN487	-18.10	-17.37	-35.47
**SQ4**	GLY416-LYS417-ILE418-ALA419	-57.72	-1.50	-59.23
**SQ5**	GLY446-GLY447-ASN448-TYR449	-7.65	-3.60	-11.26
**SQ6**	GLY502-VAL503-GLY504-TYR505	$+5.62$	-15.00	-9.38
**SQ7**	TYR453-ARG454-LEU455-PHE456	$+49.03$	-17.51	$+31.52$
**SQ8**	TYR473-GLN474-ALA475-GLY476	$+24.51$	-5.32	$+19.18$
**SQ9**	TYR489-PHE490-PRO491-LEU492	$+66.92$	-8.25	$+58.66$

[a] DFT energies computed at 6-31+G*/B3LYP level; *Dispersion* (DD) corrections evaluated with B3LYP-DD semiempirical method. [1]

[b] hACE2 residues within 4.5 Å of S-RBD *quartet* non-hydrogen atoms included.

TABLE S7. **SARS-CoV-2 S-RBD *quartets* and their interaction energies [kcal/mol][a] with neighboring[b] hACE2 residues.**

Quartet	**SARS-CoV-2 S-RBD Residues**	E_{Int}^{DFT}	E_{Int}^{DD}	E_{Int}^{Total}
**SQ1**	GLN493-SER494-TYR495-GLY496	+56.84	-7.33	+49.51
**SQ2**	GLN498-PRO499-THR500-ASN501	+5.94	-18.93	-12.99
**SQ3**	GLU484-GLY485-PHE486-ASN487	-21.07	-15.72	-36.79
**SQ4**	GLY416-LYS417-ILE418-ALA419	-60.01	-1.31	-61.32
**SQ5**	GLY446-GLY447-ASN448-TYR449	-10.23	-3.31	-13.54
**SQ6**	GLY502-VAL503-GLY504-TYR505	+3.32	-13.72	-10.40
**SQ7**	TYR453-ARG454-LEU455-PHE456	+46.11	-16.32	+29.79
**SQ8**	TYR473-GLN474-ALA475-GLY476	+23.45	-5.24	+18.21
**SQ9**	TYR489-PHE490-PRO491-LEU492	+66.68	-7.98	+58.69

[a] DFT energies computed at 6-311G(d,p)/B3LYP level; *Dispersion* (DD) corrections evaluated with B3LYP-DD semiempirical method. [1]

[b] hACE2 residues within 4.5 Å of S-RBD *quartet* non-hydrogen atoms included.

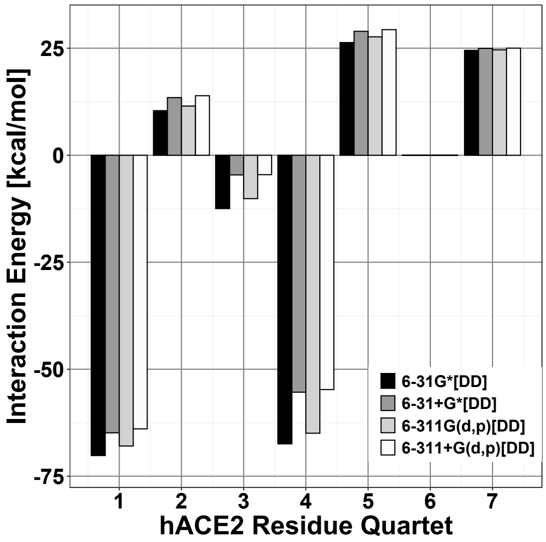

FIG. S1. **Interaction energies between hACE2 *quartets* and neighboring SARS-CoV-2 S-RBD residues.** Interactions of repulsive (positive) and attractive (negative) character [kcal/mol] between hACE2 *quartets* and SARS-CoV-2 S-RBD residues within a 4.5Å radius. For each *quartet* four adjacent vertical bars are shown corresponding to density functional calculations with 6-31G*, 6-31+G*, 6-311G(d,p) and 6-311+G(d,p) basis sets, respectively, plus additional van der Waals *dispersion* [DD] [1] corrections. Results from different basis sets are qualitatively similar. *Quartet* and *supermolecular fragment* coordinates used in calculations taken from PDB entry 6M0J.

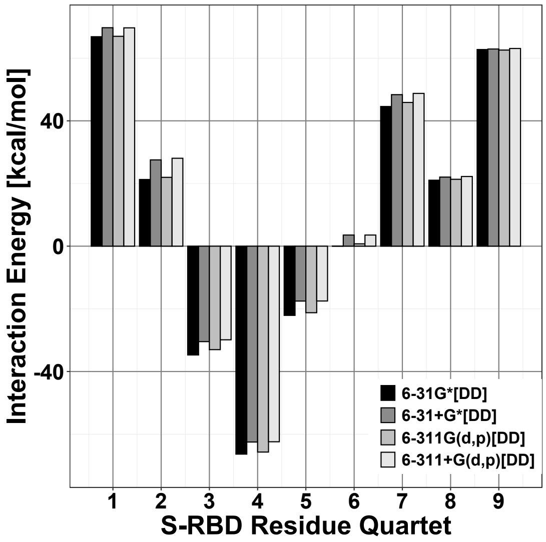

FIG. S2. **Interaction energies between SARS-CoV-2 S-RBD *quartets* and neighboring hACE2 residues.** Interactions of repulsive (positive) and attractive (negative) character [kcal/mol] between SARS-CoV-2 *quartets* and hACE2 residues within a 4.5Å radius. For each *quartet* four adjacent vertical bars are shown corresponding to density functional calculations with 6-31G*, 6-31+G*, 6-311G(d,p) and 6-311+G(d,p) basis sets, respectively, plus additional van der Waals *dispersion* [DD] [1] corrections. Results from different basis sets are qualitatively similar. *Quartet* and *supermolecular fragment* coordinates used in calculations taken from PDB entry 6M0J.

TABLE S8. Partial list of hACE2 residues making contact with the S-RBDs of SARS-CoV-1 [3] and/or SARS-CoV-2. [4]

GLN24	THR27	LYS31	HIS34	GLU37	ASP38	TYR41	GLN42	LEU45
LEU79	MET82	TYR83	ASN90	GLN325	GLU329	ASN330	LYS353	GLY354

TABLE S9. Partial list of SARS-CoV-2 S-RBD residues making contact with hACE2. [4]

LYS417	GLY446	TYR449	TYR453	LEU455	PHE456	TYR473	GLY476	ALA475	GLU484
PHE486	ASN487	TYR489	PHE490	GLN493	GLY496	GLN498	ASN501	GLY502	TYR505

REFERENCES

[1] Deligkaris, C. & Rodriguez, J. H. Correction to DFT interaction energies by an empirical dispersion term valid for a range of intermolecular distances. *Phys. Chem. Chem. Phys.* **14**, 3414–3424 (2012).

[2] Stewart, J. J. P. Optimization of parameters for semiempirical methods V: Modification of NDDO approximations and application to 70 elements. *Journal of Molecular Modeling* **13**, 1173–1213 (2007).

[3] Li, F., Li, W., Farzan, M. & Harrison, S. C. Structure of SARS coronavirus spike receptor-binding domain complexed with receptor. *Science* **309**, 1864–1868 (2005).

[4] Wang, Q. *et al.* Structural and functional basis of SARS-CoV-2 entry by using human ACE2. *Cell* **181**, 894–904 (2020).
